# The complete mitochondrial DNA sequence of the Chinese red pika (*Ochotona erythrotis*)

**DOI:** 10.1080/23802359.2018.1450663

**Published:** 2018-09-10

**Authors:** Yu-Jiao He, Yong-Gui Ma, Gong-Hua Lin, Tong-Zuo Zhang, Jian-Ping Su

**Affiliations:** a Key Laboratory of Medicinal Plant and Animal Resources in Qinghai-Tibetan Plateau, College of Life Science, Qinghai Normal University, Xining, Qinghai, P.R. China;; b Qinghai Provincial Key Laboratory of Animal Ecological Genomics, Northwest Institute of Plateau Biology, Chinese Academy of Sciences, Xining, Qinghai, P.R. China

**Keywords:** Mitochondrial genome, *Ochotona erythrotis*, phylogenetic relationship

## Abstract

In this study, we undertook the first complete *Ochotona erythrotis* mitochondrial genome. The genome sequence was 16,663 bp in length, including the typical structure of 22 transfer RNA genes, 13 protein-coding genes, 2 ribosomal RNA genes, and the non-coding control region. The overall base composition of *O. erythrotis* mitogenome is 31.8% A, 26.0% T, 28.6% G, and 13.6% C, with a high A + T content of 57.8%.

The Chinese red pika (*Ochotona erythrotis*) has the classification of *Ochotona*, Ochotonidae, Lagomorpha, Glires. This species is mainly distributed in the Qaidam Basin, Eastern Qinghai, and southwestern GanSu in China. They prefer to live in rocky terrains at altitudes above 2000 meters (Niu et al. 2001). The Chinese red pika has been little studied, especially at a molecular level. The molecular information could be used to study species classification, origin and evolution history (Liu et al. 2017). In this study, we investigated the mitochondrial genome of *O. erythrotis*. The specimen of *O. erythrotis* was collected from Huzhu, Qinghai, China, in 2015 and preserved in the key laboratory of medicinal plant and animal resources in Qinghai-Tibetan Plateau (Qinghai Normal University). The mitochondrial genome was amplified using 26 pairs of primers, based on the mitochondrial genome sequence of the plateau pika (*Ochotona curzoniae*, accession number NC_011029.1).

The complete mitochondrial genome of Chinese red pika (GenBank accession number MG_051346) was 16663 base pairs (bp). The whole mitochondrial genome coincided with many other vertebrate animals (Shan and Liu 2015; Xu et al. 2015) and contained 22 transfer RNA genes, 2 ribosomal RNA genes, 13 protein-coding genes, two main non-coding regions(the control region and the light strand replication origin). There were 8 reading frame overlaps (sharing 1–46 nucleotides) and 12 intergenic spacers (ranging from 1 to 34 bp). Its structure and composition was also comparable to other pikas (Luo et al. 2013). The total base composition was 31.8% A, 26.0% T, 28.6% C, and 13.6% G, with a slight A-T (57.8%) bias. The total length of the 13 protein-coding genes was 11,392 bp. Ten of the 13 protein-coding genes initiated with ATG, whereas *ND2* and *ND5* started with ATT and *ND3* utilized ATA. The open reading frame (ORFs) of six protein-coding genes ended with TAG or TAA, whereas *cytb* ended with AGG, and *ND6* terminated with AGA. Incomplete stop codons (T–) were found in *COX3, ND3, ND4L, ND4,* and *ND5*. The 22 tRNA genes of Chinese red pika ranged in size from59 bp (*tRNA^Ser^*) to 75 bp (*tRNA^Leu^*). The *12S* and *16S rRNA* genes were 961 bp and 1576 bp in length, respectively. The ribosomal subunit genes were located between the *tRNA^Phe^* and *tRNA^Leu^*genes and were further separated by the *tRNA^Val^*gene. The control region of the Chinese red pika mitochondrial DNA was located between the *tRNA^Pro^* and *tRNA^Phe^* genes and measured 1230 bp.

We used the mitochondrial genome of *O. erythrotis* and 15 other species of the order Lagomorpha from GenBank to construct the phylogenetic tree. *Rattus rattus* was used as outgroup for tree rooting. We used RaxmlHPC to reconstruct the phylogenetic relationship based on the maximum likelihood (ML) method (Figure 1). Analysis of the results showed that the ingroups were divided into two clades, Leporidae and Ochotonidae. *O. erythrotis* formed a single subbranch with the high bootstrap support value of 100. In previous studies, this species was placed in the subgenus *Conothoa* (Liu et al. 2017).

**Figure 1.**
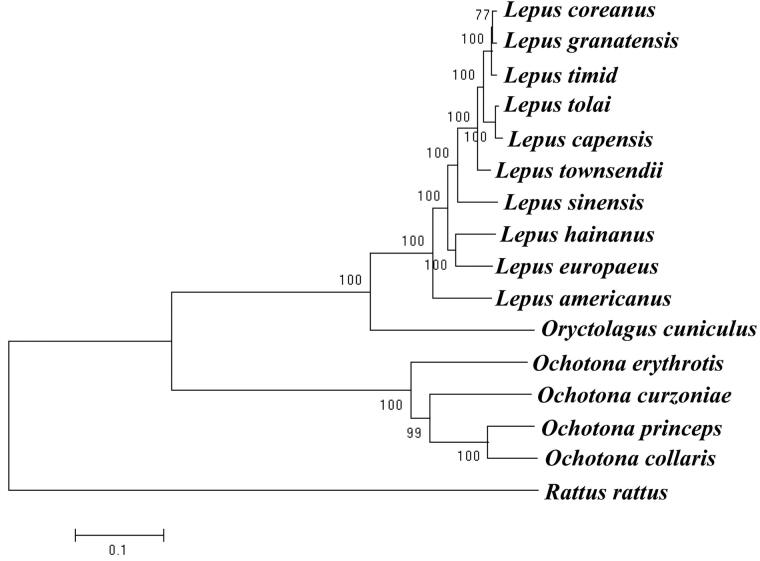
Maximum-likelihood tree of the complete mitochondrial genome of *O. erythrotis* and 15 other species. Numbers above or below the branch are bootstrap support values. The GenBank accession number for tree construction is listed as follows: *O. erythrotis* (MG_051346), *O. curzoniae* (EF535828.1), *O. princeps* (NC_005358.1), *O. collaris* (AF348080.1), *L. coreanus* (NC_024259.1), *L. granatensis* (NC_024042.1), *L. timidus* (KR019013.1), *L. tolai* (NC_025748.1), *L. capensis* (GU937113.1), *L. townsendii* (NC_024041.1), *L. sinensis* (NC_025316.1), *L. hainanus* (JQ219662.1), *L. europaeus* (KY211032.1), *L. californicus* (KJ397614.1), *L. americanus* (NC_024043.1), *O. cuniculus* (AJ001558.1), *R. rattus* (NC_012374.1). 177 × 82 mm (72 × 72 DPI)
